# Transmission of Severe Acute Respiratory Syndrome Coronavirus 2 in Households with Children, Southwest Germany, May–August 2020

**DOI:** 10.3201/eid2712.210978

**Published:** 2021-12

**Authors:** Maximilian Stich, Roland Elling, Hanna Renk, Aleš Janda, Sven F. Garbade, Barbara Müller, Hans-Georg Kräusslich, Dorit Fabricius, Maria Zernickel, Peter Meissner, Daniela Huzly, Jürgen Grulich-Henn, Anneke Haddad, Tessa Görne, Benedikt Spielberger, Linus Fritsch, Alexandra Nieters, Hartmut Hengel, Andrea N. Dietz, Thomas Stamminger, Tina Ganzenmueller, Natalia Ruetalo, Andreas Peter, Jonathan Remppis, Thomas Iftner, Kathrin Jeltsch, Tim Waterboer, Axel R. Franz, Georg Friedrich Hoffmann, Corinna Engel, Klaus-Michael Debatin, Burkhard Tönshoff, Philipp Henneke

**Affiliations:** Heidelberg University Hospital, Heidelberg, Germany (M. Stich, S.F. Garbade, B. Müller, H.-G. Kräusslich, J. Grulich-Henn, K. Jeltsch, G.F. Hoffmann, B. Tönshoff);; University Medical Centre and Faculty of Medicine Freiburg, Freiburg, Germany (R. Elling, D. Huzly, A. Haddad, T. Görne, B. Spielberger, L. Fritsch, A. Nieters, H. Hengel, P. Henneke);; University Hospital and Faculty of Medicine Tübingen, Tübingen, Germany (H. Renk, T. Ganzenmueller, N. Ruetalo, A. Peter, J. Remppis, T. Iftner, A.R. Franz, C. Engel);; Ulm University Medical Center, Ulm, Germany (A. Janda, D. Fabricius, M. Zernickel, P. Meissner, A.N. Dietz, T. Stamminger, K.-M. Debatin);; German Cancer Research Center (DKFZ), Heidelberg (T. Waterboer)

**Keywords:** COVID-19, coronavirus disease, SARS-CoV-2, severe acute respiratory syndrome coronavirus 2, viruses, respiratory infections, zoonoses, transmission, households, children, serology, antibodies

## Abstract

Resolving the role of severe acute respiratory syndrome coronavirus 2 (SARS-CoV-2) transmission in households with members from different generations is crucial for containing the current pandemic. We conducted a large-scale, multicenter, cross-sectional seroepidemiologic household transmission study in southwest Germany during May 11–August 1, 2020. We included 1,625 study participants from 405 households that each had ≥1 child and 1 reverse transcription PCR–confirmed SARS-CoV-2–infected index case-patient. The overall secondary attack rate was 31.6% and was significantly higher in exposed adults (37.5%) than in children (24.6%–29.2%; p = <0.015); the rate was also significantly higher when the index case-patient was >60 years of age (72.9%; p = 0.039). Other risk factors for infectiousness of the index case-patient were SARS-CoV-2–seropositivity (odds ratio [OR] 27.8, 95% CI 8.26–93.5), fever (OR 1.93, 95% CI 1.14–3.31), and cough (OR 2.07, 95% CI 1.21–3.53). Secondary infections in household contacts generate a substantial disease burden.

Severe acute respiratory syndrome coronavirus 2 (SARS-CoV-2) has rapidly spread globally since its emergence in December 2019. As of March 2021, >120 million infections have been reported, and >2.7 million deaths have been attributed to the novel coronavirus disease (COVID-19) (*1*). The severity of COVID-19 and the risk for a complicated course of illness or death increase with age (*2*,*3*). In terms of SARS-CoV-2 transmission, conjectures early in the pandemic were that asymptomatic (i.e., healthy) but infectious children played a particularly substantial role. The underlying assumption that children were drivers of the pandemic was based on experience with seasonal influenza virus. Consequently, closures of schools and preschools were among the earliest nonpharmaceutical interventions for transmission (*4*). However, the role of children in the transmission of SARS-CoV-2 remains controversial (*5*–*7*).

Next to superspreading events (*8*), intrahousehold transmission of SARS-CoV-2 is a major driver of the pandemic (*9*). A recent systematic review and meta-analysis based on reverse transcription PCR (RT-PCR) testing of SARS-CoV-2 RNA from nasopharyngeal or oropharyngeal swab specimens calculated a secondary attack rate (SAR) of 16.6% in households (*10*). In individual studies, the SAR in children varied from 4% (*11*) to 36% (*12*); hence, the data vary widely. Only a minority of studies reported separate SARs from pediatric index cases, and children accounted for <10% of index cases when reported (*8*,*9*,*13*,*14*).

Low detection rates of SARS-CoV-2 RNA by RT-PCR in children might not precisely reflect the frequency of infections. Mild or even asymptomatic disease in children combined with higher rates of aversion and incorrect swab collection might lead to underestimation of the infection risk, especially in symptom-based transmission studies. Determining the presence of SARS-CoV-2 antibodies could overcome some of these limitations (*15*). In a cross-sectional investigation of 2,482 child–parent pairs without known prior SARS-CoV-2 infection, we found a 3-fold lower SARS-CoV-2 seroprevalence in children than in their parents (*16*). Previous household transmission studies found SARS-CoV-2–specific IgG in 28% (*17*), 34% (*18*), 42% (*19*), 45% (*20*) and 52% (*21*) of exposed children; SARs were lower (*18*), similar to (*17*,*21*), or higher (*19*) than in exposed adult household members. However, the low number of studied households with children (21–130 households) (*17*–*21*) was a limitation.

We performed a large-scale multicenter seroepidemiologic study on transmission of SARS-CoV-2 in households with >1 child. Our objectives were to determine the SARS-CoV-2 seroprevalence and SAR in children compared with adults from the same households and, second, to identify risk factors associated with infectiousness of index case-patients and susceptibility of contacts.

## Methods

### Study Design and Conduct

We conducted a multicenter, cross-sectional SARS-CoV-2 transmission study on the prevalence of SARS-CoV-2 antibodies in members of households with 1 index case-patient with a previous SARS-CoV-2 infection confirmed by RT-PCR from a nasopharyngeal or oropharyngeal swab specimen. Households that met the eligibility criteria were invited to participate through the local health authorities Alb-Donau, Breisgau-Hochschwarzwald, Heidelberg/Rhein-Neckar, Karlsruhe, Mannheim, Neckar-Odenwald, Reutlingen, and Tübingen in the Federal State of Baden-Württemberg, Germany. We enrolled participants at the University Children’s Hospitals in Freiburg, Heidelberg, Tübingen, and Ulm during May 11–August 1, 2020. At time of study enrollment, we collected blood samples from participants for antibody measurement and retrospectively determined symptom and infection history through a questionnaire and serologic tests. The study was designed, analyzed, and reported according to the Strengthening the Reporting of Observational Studies in Epidemiology (STROBE) reporting guidelines (https://www.strobe-statement.org).

### Ethics

The study protocol was approved by the independent ethics committees of the Medical Faculty Heidelberg (approval no. S-294/2020), Medical Faculty Tübingen (approval no. 293/2020BO2), University of Ulm (approval no. 152/20), and University of Freiburg (approval no. 256/20_201553). The study was conducted according to the Declaration of Helsinki. Written informed consent was obtained from all household members and parents or guardians; children gave consent when appropriate for their age.

### Eligibility Criteria and Study Procedure

Households were eligible for enrollment if they met all of these inclusion criteria: SARS-CoV-2 detection by RT-PCR from a nasopharyngeal or oropharyngeal swab specimen in >1 household member, >1 household member <18 years of age, residency in the state of Baden-Württemberg, and all household members having been officially released from quarantine. Key exclusion criteria were lack of written consent and insufficient knowledge of the German language.

Questionnaire items were number of household members and, for each member, age, sex, and whether they had ever tested positive for SARS-CoV-2. We asked participants reporting an RT-PCR-confirmed SARS-CoV-2 infection for the date when the positive specimen was collected, COVID-19–related symptoms (fever, cough, diarrhea, or dysgeusia), and whether they were hospitalized for COVID-19. We defined the index case-patient as the household member with the first SARS-CoV-2 RNA–positive specimen collected. We validated this definition in a subset of 54 households from 1 study center for which additional questionnaire information on transmission routes from nonhousehold contacts with COVID-19 were available. In 52 (96.3%) of 54 households, the definition of the index case based on timing of the RT-PCR test was consistent with the definition based on this anamnestic information. The RT-PCR test was performed within 24 hours, and a positive test result immediately triggered a strict home isolation and quarantine for all household members for >14 days unless hospitalization was required.

### Laboratory Analysis

We sent blood samples to the respective diagnostic laboratories in the 4 study centers, and serum was prepared on the same day. Samples were either immediately analyzed or stored at 4°C until further processing. Samples were analyzed for IgG reactive to the S1 domain of the viral spike glycoprotein and the SARS-CoV-2 nucleocapsid (N) protein. Antibodies reactive to the N protein were measured either with the Elecsys Anti-SARS-CoV-2 IgG/IgM ECLIA test kit (Roche, https://www.roche.com) processed on a Roche Cobas e601 or e411 module (in Heidelberg, Tübingen, and Ulm), or by recomWell SARS-CoV-2 IgG ELISA (Mikrogen Diagnostik, https://www.mikrogen.de/start.html) run on a BEP III analyzer in (Freiburg). SARS-CoV-2 IgG for the S1 domain of the spike protein were measured with the Euroimmun Anti-SARS-CoV-2-ELISA (IgG) test kit (Euroimmun, https://www.euroimmun.com) in Freiburg and Ulm. In Heidelberg and Tübingen, IgG/IgM directed against the receptor-binding domain of S1 were analyzed with the SARS-CoV-2 Total (COV2T) CLIA Assay (Siemens Healthineers, https://www.siemens-healthineers.com) on a Siemens ADVIA Centaur XP analyzer.

We categorized serum samples with concordant results in both assays as seropositive or seronegative. In case of discordant results, we performed additional, study site–specific measurements. These measurements were a neutralization assay (Tübingen) (*22*); the Euroimmun Anti-SARS-CoV-2-ELISA (IgG) (Euroimmun) (Heidelberg); the Elecsys Anti-SARS-CoV-2 IgG/IgM ECLIA (Roche) (Freiburg); or the ARCHITECT SARS-CoV-2 IgG, a test for IgG against the viral N protein (Abbott Laboratories, https://www.abbott.com) on an Abbott ARCHITECT 1000 instrument (Ulm). We classified serum samples with a positive reaction in the additional assay as seropositive.

### Statistical Analysis

We performed analyses with R version 4.0.0 (R Foundation for Statistical Computing, https://www.r-project.org). We present results for continuous variables as mean with SD (for data with normal distribution) or median with interquartile ranges (IQR) and minimum and maximum values, unless stated otherwise. SARS-CoV-2 seropositivity served as a proxy for previous infection. We calculated the observed SAR by dividing the number of exposed SARS-CoV-2 IgG-positive household members by all exposed household members. To model and predict SAR, we used generalized linear mixed-effects logistic regression models (GLMM) with a logit function and the dependent variable “SARS-CoV-2 infection (yes/no)” of exposed household members and the predictors age of index case-patient, age of exposed household member, sex of index case-patient, sex of exposed household member, household size, and SARS-CoV-2–seropositivity in the index case.

We used a generalized linear mixed-effects model tree (*23*) to detect subgroup interactions in SAR of exposed household members (R package glmertree). This method uses model-based recursive partitioning to detect subgroup interactions and a GLMM to estimate the random-effects parameters (*23*). No a priori formulated hypotheses were tested, and therefore all p values and CIs are reported as descriptive measures. We compiled a more detailed description of GLMM models, simulations, violin plots, and R code (Appendix)

## Results

### Study Population

**Figure 1:**
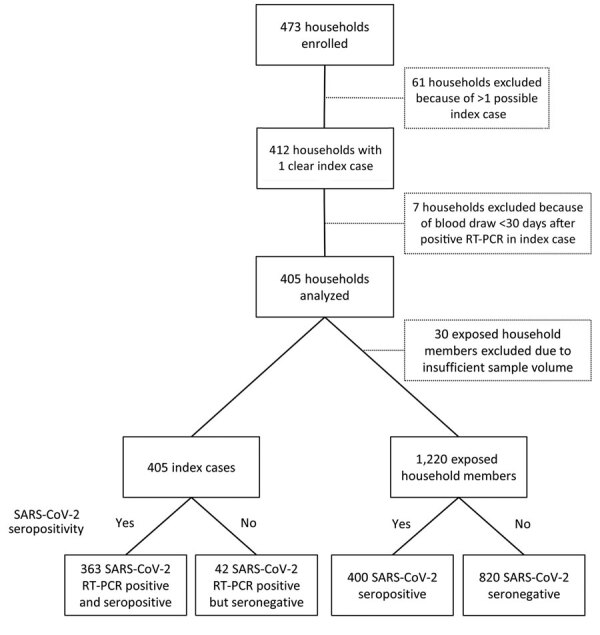
Flowchart of participant enrollment in study of transmission of severe acute respiratory syndrome coronavirus 2 in households with children, southwest Germany, May–August 2020. RT-PCR, reverse transcription PCR; SARS-CoV-2, severe acute respiratory syndrome coronavirus 2.

We enrolled 473 households during May 11–August 1, 2020 (Appendix Figure). We excluded households in which the index case could not be determined (n = 61). SARS-CoV-2–seropositivity plateaued at ≈30 days after a positive RT-PCR test for SARS-CoV-2 RNA (Appendix Table 1). To reduce the probability of negative serologic results because of imminent seroconversion, we excluded households that participated <30 days after a positive RT-PCR test of the index case (n = 7). A total of 405 households with 1,625 members (922 adults and 703 children) were available for final analysis (Table 1; Figure 1). The median age of index case-patients (n = 405) was 43.6 (range 1.36–71.5) years; 25 index case-patients (6.2%) were children. Among exposed household members (n = 1,220), 678 participants (55.6%) were children and 542 (44.4%) were adults. The sex distribution of index case-patients and exposed household members was balanced (Table 1).

**Table 1:** Demographic characteristics of study participants from 405 households, southwest Germany, May–August 2020

Characteristic	Total cohort	Adults	Children
No. participants	1,625	922	703
Median age, y	30.0	42.6	10.0
Interquartile range	11.0–45.0	37.0–50.0	5.79–13.9
Range	0.50–81.1	18.0–81.1	0.50–17.9
No. index case-patients	405	380	25
Median age, y	43.6	44.8	13.3
Interquartile range	37.2–49.5	38.0–49.9	9.03–16.2
Range	1.36–71.5	18.3–71.5	1.36–17.6
No. exposed household members	1220	542	678
Median age, y	16.2	42.8	9.83
Interquartile range	8.99–41.0	35.4–50.0	5.58–13.8
Range	0.50–81.1	18.0–81.1	0.50–17.9
Sex			
M	807	457	350
F	818	465	353
Household size*			
2–3	267	174	93
4	804	449	355
5	360	192	168
>6	194	107	87
Region			
Freiburg	577	329	248
Heidelberg	532	306	226
Tübingen	319	175	144
Ulm	197	112	85

*Includes household members who did not participate.

### SARS-CoV-2 Seropositivity and Observed Secondary Attack Rates

A total of 400 of 1,220 exposed household members tested positive for SARS-CoV-2 IgG and were categorized as previously infected (Figure 1), resulting in an overall observed SAR of 32.8%. Among the 405 index case-patients with RT-PCR–confirmed SARS-CoV-2 infection, 363 (89.6%) were seropositive and 42 (10.4%) were seronegative at the time of study participation. The rate of seropositivity in households with a seropositive index case-patient (393 of 1,090 [36.1%]) was 6-fold higher than the rate in households with a seronegative index case-patient (7 of 130 [5.4%]) (Table 2). The observed SAR in adults was 38.0% (206 of 542) compared with 28.6% (194 of 678) in children; it did not differ substantially among the 3 pediatric age groups (<6 years, 26.6%; 6–11.9 years, 30.7%; 12.0–17.9 years, 27.9%).

**Table 2:** Secondary attack rates in household members exposed to severe acute respiratory syndrome coronavirus 2 from 405 households, southwest Germany, May–August 2020*

Characteristic	No. index cases	No. exposed	No. seropositive exposed	Observed SAR, %	Predicted SAR, % (IQR)†	Odds ratio (95% CI)	p value
No. participants	405	1,220	400	32.8	31.6 (8.31–52.2)	NA	NA
Age of index case-patients, y							
>60	6	21	15	71.4	72.9 (54.9–88.9)	9.02 (1.19–72.8)	0.039
18.0–59.9	374	1122	366	32.6	31.3 (8.41–51.1)	Referent	
12.0–17.9	16	47	15	31.9	30.8 (3.11–55.9)	1.32 (0.31–5.57)	0.704
0.0–11.9	9	30	4	13.3	12.0 (0.59–11.4)	0.34 (0.04–3.19)	0.343
Age of exposed household members, y							
>18	NA	542	206	38.0	37.5 (13.2–59.4)	Referent	
12.0–17.9	NA	244	68	27.9	25.8 (6.24–40.2)	0.39 (0.25–0.63)	<0.001
6.0–11.9	NA	257	79	30.7	29.2 (8.02–47.9)	0.55 (0.35–0.89)	0.015
0.0–5.9	NA	177	47	26.6	24.6 (5.09–43.8)	0.33 (0.18–0.58)	<0.001
Sex of index case-patients							
M	207	629	213	33.9	32.6 (8.52–53.6)	Referent	
F	198	591	187	31.6	30.4 (8.12–50.8)	1.07 (0.62–1.87)	0.803
Sex of exposed household members							
M	NA	600	195	32.5	31.1 (7.62–51.9)	Referent	
F	NA	620	205	33.1	31.9 (8.54–53.5)	1.08 (0.75–1.56)	0.676
Household size							
2–3	92	175	69	39.4	38.1 (12.5–67.1)	Referent	
4	206	598	185	30.9	29.4 (8.26–44.0)	0.50 (0.24–1.01)	0.055
5	75	285	98	34.4	33.5 (8.57–53.7)	0.77 (0.33–1.78)	0.543
>6	32	162	48	29.6	28.8 (4.64–55.6)	0.40 (0.14–1.18)	0.095
SARS-CoV-2–seropositive index case-patient							
No	42	130	7	5.38	3.59 (0.71–2.17)	Referent	
Yes	363	1090	393	36.1	34.9 (12.0–56.4)	27.8 (8.26–93.5)	<0.001

*IQR, interquartile range; NA, not applicable; SAR, secondary attack rate. †The predicted SARs, odds ratios, and p values were calculated from a multivariable generalized linear mixed-effects logistic regression model. The respective references were “adult index 18.0-59.9 years,” “adult exposed,” “male index,” “male exposed,” “household size 2–3,” and “seronegative index.”

The observed SAR in exposed household members increased with the age of the index case-patient, from 13.3% for those <12 years of age to 71.4% for those >60 years of age (Table 2). The observed SAR in exposed male (32.5%) and female (33.1%) household members and in those with a male (33.9%) or female (31.6%) index case-patient were similar. Among the 405 index case-patients, 394 (97.3%) reported COVID-19–related symptoms and 9 (2.2%) were asymptomatic; no data were reported for 2 index case-patients. The most prevalent symptom was dysgeusia; cough, fever, and diarrhea were next most prevalent (Appendix Table 2). Overall symptoms (98.3% vs. 88.1%), and especially fever (58.4% vs. 33.3%) and dysgeusia (66.1% vs. 31.0%), were more prevalent in seropositive index case-patients than in seronegative index case-patients. A total of 22 (5.4%) index case-patients were hospitalized.

### Risk Factors for SARS-CoV-2 Transmission

We used a linear mixed-effects logistic regression model to analyze these risk factors for virus transmission: age and sex of index case-patients and of exposed household members, household size, and SARS-CoV-2 seropositivity of the index case-patient (Table 2). SARS-CoV-2 seropositivity of the index case-patient was the risk factor most strongly associated with the SAR (odds ratio [OR] 27.8, 95% CI 8.26–93.5; p<0.001).

The predicted SAR in adults was higher than the predicted SARs in the 3 pediatric age groups, which were broadly similar (Table 2). Age of the index case-patient was also a risk factor for virus transmission. The predicted SAR in exposed household members was lowest when the index case-patient was <12 years of age (12.0%) and highest with an index case-patient >60 years of age (72.9%) and plateaued around 31% for index case-patients 12.0–59.9 years of age. It differed significantly between adults 18.0–59.9 years of age and those >60 years of age (OR 9.02, 95% CI 1.19–72.8; p = 0.039). Sex of the index case-patient and sex of the exposed household member were not associated with the SAR (Table 2). Larger households tended toward lower predicted SARs (Table 2); when we applied a Fisher exact test to the observed data, households with >4 household members were associated with a lower SAR (Appendix Table 3).

**Figure 2:**
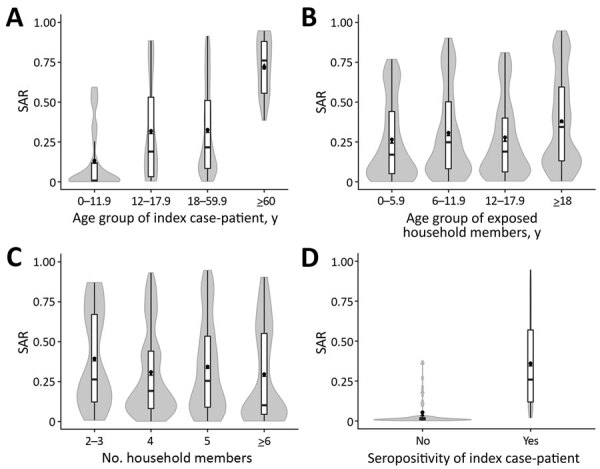
Observed and predicted SARs in household members exposed to severe acute respiratory syndrome coronavirus 2, southwest Germany, May–August 2020. SARs shown are associated with age of index case-patient (A), age of exposed household member (B), household size (C), and SARS-CoV-2 seropositivity of the index case-patient (D). The mean observed SAR is shown as a black dot. The mean (black triangles), interquartile range (white bars), maximum and minimum (ends of vertical black line), and distribution (gray shading) of the predicted SAR are shown in the violin plots. The predicted SARs were calculated from the generalized linear mixed-effects logistic regression model. SAR, secondary attack rate.

We compared the observed and predicted SAR associated with age of the index case-patient, age of exposed household members, household size, and SARS-CoV-2 seropositivity of the index case-patient (Figure 2). We calculated the predicted SAR by using the generalized mixed-effects logistic regression model with simulations. In all 4 analyses, the observed and predicted SARs were almost identical, indicating that this logistic regression model was valid.

**Figure 3:**
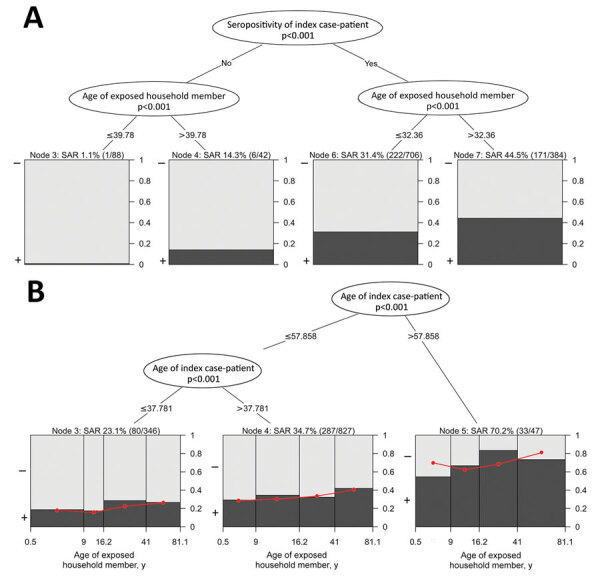
Generalized linear mixed model binary decision trees in study of transmission of severe acute respiratory syndrome coronavirus 2 in households with children, southwest Germany, May–August 2020. A) Model incorporating the 2 most dominant effects (p<0.001) on the SAR of exposed household members, SARS-CoV-2 seropositivity of the index case-patient and age of exposed household members with a seronegative or a seropositive index case-patient. B) Model incorporating only age of the index case-patient as a risk factor; SAR was modeled by age of exposed household member within each node. In both panels, the observed SAR as a proportion of seropositive (black) and seronegative (gray) exposed household members with these characteristics are shown within final nodes and as a percentage with the total number of seropositive/total exposed household members in parentheses above each node. In panel B, the predicted SARs are indicated within each final node as a red dot and red straight line. SAR, secondary attack rate.

We used the same risk factors for a generalized linear mixed model binary decision tree to study subgroup interactions of risk factors for SAR. The most dominant risk factor for transmission was SARS-CoV-2-seropositivity of the index case-patient; the next most dominant risk factor was increased age of exposed household members (Figure 3, panel A). In an alternative generalized linear mixed model binary decision tree, only age of the index case-patient was considered a risk factor, and the age of exposed household members was fixed in each terminal node (Figure 3, panel B). In this model, the SAR increased with age of the index case-patient. Within each age group of index case-patient, the SAR also increased with age of the exposed household member (Figure 3, panel B). The observed SAR was 23.1% (80/346) if the index case-patient was <37.8 years of age, 34.7% (287/827) if the index case-patient was 37.8–57.9 years of age, and 70.2% (33/47) if the index case-patient was >57.9 years of age. However, cutoff values determined by generalized linear mixed-effects model trees are data-driven and should not be interpreted as fixed parameters. When we excluded the 42 households with a seronegative index case-patient and analyzed the remaining 363 households (Appendix Table 4) or included the time interval from positive SARS-CoV-2 RNA specimen collection in the index case-patient to the serologic assessment of the household (Appendix Table 5), we obtained comparable results.

We analyzed the COVID-19–related symptoms cough, fever, dysgeusia, and diarrhea, as well as hospitalization in the index case-patient, in an additional linear mixed-effect logistic regression model, consisting only of households with a symptomatic index case-patient with known hospitalization status (n = 393) and adjusted for age of the index case-patient. The occurrences of fever and cough, but not of diarrhea, dysgeusia, or hospitalization, were significantly associated with a higher predicted SAR (Table 3).

**Table 3:** Association between coronavirus disease–related symptoms or hospitalization in index case-patients and secondary attack rates in exposed household members from 393 households with a symptomatic index case-patient whose hospitalization status is known, southwest Germany, May–August 2020*

Index case symptom or hospitalization	No. index cases, n = 393	No. exposed, n = 1182	No. seropositive exposed, n = 390	Observed SAR, %	Predicted SAR, % (IQR)†	Odds ratio (95% CI)†	p value†
Fever	225	666	253	38.0	36.8 (11.8–58.4)	1.93 (1.14–3.31)	0.015
Cough	236	717	272	37.9	36.8 (11.7–58.2)	2.07 (1.21–3.53)	0.008
Diarrhea	91	271	94	34.7	33.2 (9.34–54.5)	0.80 (0.43–1.49)	0.481
Dysgeusia	253	748	252	33.7	32.2 (10.4–48.7)	1.41 (0.82–2.43)	0.213
Hospitalization	22	67	30	44.8	43.9 (15.2–68.9)	1.22 (0.40–3.75)	0.726

*IQR, interquartile range; NA, not applicable; SAR, secondary attack rate. †The predicted SARs, odds ratios, and p values were calculated from a multivariable generalized linear mixed-effects logistic regression model. The respective references were absence of the symptom or “hospitalization” and adjusted for age of index case-patient as a numeric variable (OR 1.04, 95% CI 1.01–1.06; p = 0.0055).

## Discussion

This large multicenter serologic SARS-CoV-2 household transmission study focusing on children revealed that the predicted SAR in household members <18 years of age is ≈8–13 percentage points lower than in adults. The predicted SAR also increased with increasing age of the index case-patient, which resulted in SARs of exposed household members ranging from 12.0% when the index case-patient was <12 years of age to 72.9% when the index case-patient was >60 years of age. The infectiousness of teenagers was similar to adults <60 years of age, and the predicted SAR was 31% in both groups.

Next to age, a systemic immune response after SARS-CoV-2 infection in the index case-patient, as indicated by circulating virus-specific antibodies, was strongly associated with the occurrence of secondary household cases. The biologic basis for the strikingly low SAR of 5.4% in households with a seronegative index case-patient (42/405) is unclear. Given the high specificity of SARS-CoV-2 RT-PCR testing, a proportion of 10% false-positive results is unlikely. Presumably, the individual viral load is associated with both a stronger adaptive immune response and the extent of symptoms, which in turn increase virus transmission. Our observations that fever and dysgeusia were less prevalent in seronegative index case-patients and that presence of fever and cough in the index case-patient increases SAR in exposed household members are in line with this hypothesis. Furthermore, our findings are in accordance with other studies, in which specific SARS-CoV-2 antibodies were frequently absent in patients with mild symptoms (*15*). However, the hypothesis that SARS-CoV-2 transmission is more likely in cases with higher or persisting viral load in the nasopharynx has not been formally tested.

Our observation of a SAR ≈10 percentage points higher in adults than in children is consistent with household studies based on RT-PCR–confirmed SARS-CoV-2 infection (*10*). In contrast, previous household transmission studies based on SARS-CoV-2 serologic testing reported lower (*18*), similar (28%) (*17*), or higher (43%–52%) (*19*–*21*) SARs in children. However, these studies were relatively small.

A low proportion of pediatric index case-patients (6.2%) and an increasing SAR with increasing age of the index case-patient is in line with most previous serologic testing–based (*24*) or RT-PCR–based (*10*,*13*,*25*) household transmission studies comparing infectiousness of pediatric and adult index case-patients. In addition, Soriano-Arandes et al. (*13*) found fewer intrahousehold transmissions after reopening schools, whereas intraschool transmissions were rare events in several countries after schools reopened in 2020 (*26*–*32*). Keeping schools open with strict hygiene measures in place could reduce overall SARS-CoV-2 transmission because close intrahousehold contact is reduced and children might act as sentinels for household transmissions when regularly tested at school.

Lower SARs in children have been previously attributed to differences in contact patterns; for example, physical interactions between spouses might be more intimate than between children and adults (*33*). Accordingly, we hypothesized that among children, toddlers might have more frequent and close physical contact with their parents than older children and adolescents, which might result in a SAR inversely correlated with age. However, we found the SAR to be similar among toddlers, older children, and adolescents, which indicates that behavior might not have a major effect on virus transmission within families. In contrast, the lower susceptibility to SARS-CoV-2 in children points toward the possible role of developmental factors related to host resistance and immunity. Low expression levels of angiotensin-converting enzyme 2, the cellular entry receptor of SARS-CoV-2 in the nasal epithelium of children has been previously suggested as a mechanistic factor (*34*). Moreover, previous endemic coronavirus infections in children might provide some protection, as indicated by frequently circulating cross-reacting antibodies (*35*) and SARS-CoV-2–reactive CD4+ T cells in <60% of unexposed children and adolescents (*36*). Furthermore, the innate immune response in children with SARS-CoV-2 exposure and infection might differ from that in adults, such as with respect to circulating neutrophil subsets, the induction of interferons (*37*), and cytokines (*38*).

The strengths of this study are its multicenter design, the high number of study participants, the application of robust statistical models, and a relatively low risk for recruitment bias, because potentially eligible households were invited through health authorities. Limitations are the high proportion of symptomatic index case-patients (97%) and adult (94%) index case-patients. Potential explanations are the limited diagnostic test capacities at the time of the study, which favored testing of symptomatic adults and those with work-related exposure (e.g., in the healthcare sector) or a history of travel to high-risk regions. In households with an asymptomatic index case-patient, symptomatic secondary case-patients were possibly mislabeled as index case-patients as a result of this testing policy. An overestimation of the SAR in adults and an underestimation in children is likely, because COVID-19-associated symptoms were a good predictor for SARS-CoV-2 infection in adults but not in children (*16*). COVID-19–related symptoms were not reported more frequently in seropositive children than seronegative children, and other respiratory viruses were >100 times more prevalent than SARS-CoV-2 in children with acute respiratory symptoms across Germany during February–May 2020 (*39*). Another factor that leads to an underestimation of the SAR is a negative SARS-CoV-2 IgG test result in <10% of previously infected participants, which might partly be because of false-negative test results and a physiologic reduction of SARS-CoV-2 IgG levels over time.

Other potential weaknesses of serologic testing–based household transmission studies are the difficulty of differentiating between secondary and tertiary transmission within the same household and the inability to rule out nonhousehold infections of exposed household members. This could result in an overestimation of the SAR per index case. The possibility that 1 nonhousehold (community) index case-patient infected several household members is a potential bias of this study. However, the probability of SARS-CoV-2 infections from 2 different nonhousehold (community) index case-patients can be assumed to be low, because this study was performed shortly after the first pandemic wave and the strict government-imposed lockdown (Appendix Figure), when the SARS-CoV-2 seroprevalence in southwest Germany was as low as 1.8% in adults and 0.6% in children (*16*). Finally, the findings regarding SAR and its age dependency only apply to the SARS-CoV-2 variants circulating in Germany at that time and might not be translated to the more transmissible Delta variant.

In conclusion, this multicenter SARS-CoV-2 household transmission study focusing on children demonstrates that secondary infections in household contacts generate a substantial disease burden. Age is a risk factor both for infectiousness of index cases and susceptibility of exposed household members. Furthermore, fever and cough in index case-patients were associated with higher levels of infectiousness. Households can be expected to remain sites for SARS-CoV-2 transmission because home quarantine and home isolation are key measures in cases of suspected or confirmed infections in most countries.

Appendix Additional information about transmission of severe acute respiratory syndrome coronavirus 2 in households with children, southwest Germany, May–August 2020
