# Supplementary material for: Transmission of Severe Acute Respiratory Syndrome Coronavirus 2 in Households with Children, Southwest Germany, May–August 2020
Source: Emerg Infect Dis. 2021 Dec;27(12):3009–19. doi: 10.3201/eid2712.210978 (PMC8632156; doi:10.3201/eid2712.210978)
Supplement: Appendix — Additional information about transmission of severe acute respiratory syndrome coronavirus 2 in households with children, southwest Germany, May–August 2020 [file 21-0978-Techapp-s1.pdf]

Article DOI: https://doi.org/10.3201/eid2712.210978

Transmission of Severe Acute Respiratory Syndrome Coronavirus 2 in Households with Children, Southwest Germany, May–August 2020

Appendix

Statistical Description of Generalized Linear Mixed Models and Predicted Secondary Attack Rates

The variable ‘household id’ was used as a random factor; thus, each household has its own intercept. We categorized the age of exposed household members (age <6 years, 6.0–11.9 years, 12.0–17.9 years, and ≥18 years) and the age of the index case-patient (age <12 years, 12.0–17.9 years, 18.0–59.9 years, and ≥60 years). The function ‘glmer’ from the package ‘lme4’ version 1.1–23 was used to fit mixed-effects logistic regression models. We used the function ‘sim’ from the package ‘arm’ (*1*) to obtain simulations to derive predicted secondary attack rates (SARs) for each study participant. Function ‘sim’ generates 1,000 sets of parameter estimates, assuming a uniform (flat) prior distribution on all parameters in generalized linear mixed models (GLMM), calculates the posterior distribution analytically, and then generates random samples from this distribution to predict individual SARs. Individual predicted SARs were visualized across covariates of interest by violin plots (R package ‘ggplot2’). We computed 95% CI for odds ratios with the function ‘confint’ by using the Wald method. We also compared the predicted SAR (outcome) of exposed household members from index case-patients with and without ≥1 COVID-19-associated symptoms (fever, cough, diarrhea, or dysgeusia) in a separate mixed-effects logistic regression model.

R Code and Description

Code Used to Generate Table 2 (Main GLMM)

Helper function to extract complete data from data frame:

```
## select complete data from a data frame
scd <- function(data, var.s, drop = TRUE, check = ”ID,” rows = 2) {
 if(!all(vars %in% colnames(data))) {
    stop(“non maching names in argument 'vars'”)
 }
 data <- data[, var.s]
 data <- na.omit(data)
 ## select only
 if(is.character(check) and is.numeric(rows)) {
    data <- do.call(“rbind,” lapply(split(data, data[, check]), function(x) {
        if(nrow(x)> = rows)
            return(x)
    }))
 }

 if(drop)
    data <- droplevels(data)
 return(invisible(data))
}
```

Select complete data from database:

```
ss <- scd(expo, c(“covid19,” “antibody,””age.ipat4,””hhs2,” “hhs,”
              “sexi,””sexe,” “AgeGroup,”
              “FID”),
         check = NULL)
```

We used library ‘lme4’ to fit GLMM:

```
require(lme4)
fit0 <- glmer(covid19 ~ antibody + hhs2 + age.ipat4 +sexi + sexe+
        AgeGroup + (1|FID), data = ss,
  control = glmerControl(optimizer = ”bobyqa,”optCtrl = list(maxfun = 2e5)),
        family = binomial)
```

Odds ratios with Wald confidence intervals can be computed with:

```
>exp(cbind(odds = fixef(fit0), confint(fit0, parm = ”β_,”method = ”Wald”)))
```

We draw simulations from fitted GLMM with the sim() function from package ‘arm’. This function assumes a uniform (flat) prior distribution on all parameters, calculates the posterior distribution analytically, and then generates random samples from this distribution. The package ‘arm’ is available on GitHub (https://github.com/cran/arm).

```
## draw simulations
require(arm)
PI.arm.sims <- arm::sim(fit0, 1000)
PI.arm <- data.frame(
 fit = apply(fitted(PI.arm.sims, fit0), 1, function(x)
        quantile(x, 0.500)),
 upr = apply(fitted(PI.arm.sims, fit0), 1, function(x)
        quantile(x, 0.975)),
 lwr = apply(fitted(PI.arm.sims, fit0), 1, function(x)
        quantile(x, 0.025))
)
## combine simulations of predicted values with observed data for further ##
visualizations.
```

s2 <- data.frame(ss, PI.arm)

Code Used to Generate Table 3 (GLMM with Symptoms of Index Case-Patient)

Select data: only households with index patients with known reported symptoms:

expo.bak <- expo

i <- with(sar, index.pat = = “I” & V13_symptome_covid = = “ja”)

ids <- unique(sar$v1_code_familie[i])

expo2 <- subset(expo, FID %in% ids)

Select complete data:

s <- scd(expo2, var.s = c(“covid19,” “Husten,” “Fieber,” “Durchfall,”

“Geschmack,” “age.ipat,” “hospi.ipat,” “FID”), check = NULL)

Fit GLMM:

fit <- glmer(covid19 ~ Husten+Fieber+Durchfall+Geschmack + age.ipat +

hospi.ipat + (1|FID), data = s,

control = glmerControl(optimizer = ”bobyqa,”optCtrl = list(maxfun = 2e5)),

family = binomial)

Odds ratios and confidence intervals:

>exp(cbind(Est = fixef(fit), confint(fit, parm = ”β_,” method = ”Wald”)))

Code Used to Generate Figure 2, Panel A (Age of Index Case-Patient)

Note: s2 is a data frame with raw data and predicted SAR, see code above (Main GLMM).

m2 <- aggregate(fit ~ age.ipat4, data = s2,

FUN = ”mean”)

f1 <- xtabs(~ covid19 + age.ipat4, data = ss)

fp <- f1[2,]/colSums(f1)

fp2 <- as.data.frame(fp)

```
fp2 <- data.frame(fp, hhs2 = rownames(fp2))

require(ggplot2)

p < - ggplot(s2, aes(x = age.ipat4, y = fit)) +
  geom_violin(col = ”gray,” fill = ”lightgray”)+
  geom_boxplot(width = 0.1, outlier.shape = NA) +
  ##geom_boxplot(fill = ”lightgray”) +
  geom_point(data = fp2, aes(x = age.ipat4, y = fp), size = 1.2) +
  geom_point(data = m2, aes(x = age.ipat4, y = fit), shape = 17, size = 1.2) +
  theme_bw() + ylim(c(0, 1)) +
  theme(axis.text.x = element_text(angle = 0, hjust = 0.5),
        legend.position = “bottom”) +
  ## change x-ticks expression(phantom()> = 18)))
  scale_x_discrete(labels = c(“[0,12)” = ”0–11.9,”
                              “[12,18)” = ”12–17.9,”
                              “[18,60)” = ”18–59.9,”
                              “[60,Inf)” = expression(phantom() > = 60))) +
  labs(x = “Age groups (years) of index case,” y = “Secondary attack rate”)

p
```

Code Used to Generate Figure 2, Panel B (Age of Exposed Household Member)

```
m2 <- aggregate(fit ~ AgeGroup, data = s2,
           FUN = ”mean”)

f1 <- xtabs(~ covid19 + AgeGroup, data = ss)

fp <-  f1[2,]/colSums(f1)

fp2 <- as.data.frame(fp)
```

```
fp2 <- data.frame(fp, hhs2 = rownames(fp2))
p < - ggplot(s2, aes(x = AgeGroup, y = fit)) +
  geom_violin(col = "gray," fill = "lightgray")+
  geom_boxplot(width = 0.1) +
  ##geom_boxplot(fill = "lightgray") +
  geom_point(data = fp2, aes(x = Group, y = fp), size = 1.2) +
  geom_point(data = m2, aes(x = AgeGroup, y = fit), shape = 17, size = 1.2) +
  theme_bw() + ylim(c(0, 1)) +
  theme(axis.text.x = element_text(angle = 0, hjust = 0.5),
        legend.position = "bottom") +
  ## change x-ticks expression(phantom()> = 18)))
  scale_x_discrete(labels = c("[0,6)" = "0–5.9,"
                              "[6,12)" = "6-11.9,"
                              "[12,18)" = "12–17.9,"
                              "[18,Inf]" = expression(phantom() > = 18))) +
  labs(x = "Age groups (years) of exposed household members," y = "Secondary attack
rate")
p
```

Code Used to Generate Figure 2, Panel C (Household Size)

```
m2 <- aggregate(fit ~ hhs2, data = s2,
            FUN = "mean")
f1 <- xtabs(~ covid19 + hhs2, data = ss)
fp <-  f1[2,]/colSums(f1)
fp2 <- as.data.frame(fp)
fp2 <- data.frame(fp, hhs2 = rownames(fp2))
```

```
p < - ggplot(s2, aes(x = hhs2, y = fit)) +
  geom_violin(col = ”gray,” fill = ”lightgray”)+
  geom_boxplot(width = 0.1) +
  ##geom_boxplot(fill = ”lightgray”) +
  geom_point(data = fp2, aes(x = hhs2, y = fp), size = 1.2) +
  geom_point(data = m2, aes(x = hhs2, y = fit), shape = 17, size = 1.2) +
  theme_bw() + ylim(c(0, 1)) +
  theme(axis.text.x = element_text(angle = 0, hjust = 0.5),
        legend.position = “bottom”) +
  labs(x = “Number of household members,” y = “Secondary attack rate”) +
  scale_x_discrete(labels = c(“6+” = expression(phantom() > = 6)))
p
```

Code Used to Generate Figure 2, Panel D (SARS-CoV-2 Seropositivity of the Index Case-Patient)

```
m2 <- aggregate(fit ~ antibody, data = s2,
            FUN = ”mean”)
f1 <- xtabs(~ covid19 + antibody, data = ss)
fp <-  f1[2,]/colSums(f1)
fp2 <- as.data.frame(fp)
fp2 <- data.frame(fp, hhs2 = rownames(fp2))
p < - ggplot(s2, aes(x = antibody, y = fit)) +
  geom_violin(col = ”gray,” fill = ”lightgray”)+
  geom_boxplot(width = 0.05, outlier.shape = NA) +
  ##geom_boxplot(fill = ”lightgray”) +
  geom_point(data = fp2, aes(x = antibody, y = fp), size = 1.2) +
```

```
  geom_point(data = m2, aes(x = antibody, y = fit), shape = 17, size = 1.2) +
  theme_bw() + ylim(c(0, 1)) +
  theme(axis.text.x = element_text(angle = 0, hjust = 0.5),
        legend.position = “bottom”) +
  labs(x = “Seropositivity of index case,” y = “Secondary attack rate”)
p
```

Code Used to Generate Figure 3, Panel A

We used package ‘glmertree’ to compute GLMM Trees.

Prepare data:

```
ss <- scd(expo, c(“covid19,” “antibody,””hhs2,” “sexi,””sexe,”  “age.expo,” “FID”),
check = NULL)
Fit GLMM tree
library(glmertree)
ss3 <- ss
colnames(ss3) <- rename.cols(colnames(ss3),
                   c(“age.expo” = ”age of exposed household member,”
                     “age.ipat” = ”age of index case,”
                     “antibody” = ”seropositivity of index case”))
## fit
fit <- glmertree(covid19 ~ 1  | FID | `age of exposed household member` +
          `age of index case` +
          `seropositivity of index case` + hhs2 +
          sexi + sexe,
        cluster = FID,
        family = binomial,
```

```
data = ss3)
fit$tree
ss$Group <- predict(fit, type = ”node”)
ss3$Group <- predict(fit, type = ”node”)
```

Plot tree:

```
plot(fit)
```

Code Used to Generate Figure 3, Panel B

```
fit2 <- glmertree(covid19 ~ `age of exposed household member` | FID |
        `age of index case`,
        family = binomial, data = ss3)
```

Plot tree:

```
plot(fit2)
```

Code Used to Generate Appendix Table 3 (Fisher's Exact Test)

```
>m <- matrix(c(106,714,69,331),ncol = 2, dimnames = list(c(“2-3,””4+”),
                c(“not infected,” “infected”)))
```

Code Used to Generate Appendix Table 4 (GLMM for Sensitivity Analysis 1)

First, select households with a seropositive index case, then create some new variables:

```
## select families wihout Index case w/o symptoms
i <- with(sar, index.pat = = “I” & var_virologie = = “ja”)
ids <- unique(sar$v1_code_familie[i])
ss <- subset(expo, FID %in% ids)
ss <- droplevels(ss)
## select complete data
ss <- scd(ss, c(“covid19,” “antibody,””age.ipat,””hhs2,”
      “sexi,””sexe,” “AgeGroup,” “FID”),
```

```
        check = NULL)
ss$age.ipat4 <- cut(ss$age.ipat, breaks = c(0, 12, 18, 60, Inf),
            right = FALSE)
ss$age.ipat4 <- factor(ss$age.ipat4, levels = c(“[18,60),”
                        “[0,12),”
                        “[12,18),”
                        “[60,Inf)”))
ss$AgeGroup <- factor(ss$AgeGroup, levels = c(“[18,Inf],”
                        “[0,6),”
                        “[6,12),”
                        “[12,18)”))
```

Fit GLMM:

```
fit3 <- glmer(covid19 ~ age.ipat4 + hhs2 + sexi + sexe+AgeGroup +
            (1|FID), data = ss,
   control = glmerControl(optimizer = ”bobyqa,”optCtrl = list(maxfun = 2e5)),
            family = binomial)
```

Odds ratios and Wald confidence intervals:

```
>m <- exp(cbind(odds = fixef(fit3), confint(fit3, parm = ”β_,” method = ”Wald”)))
```

Code Used to Generate Appendix Table 5 (GLMM for Sensitivity Analysis 2)

Select data from database:

```
ss <- scd(expo, c(“covid19,” “antibody,””age.ipat,””hhs2,”
          “sexi,””sexe,” “AgeGroup,” “span.ind,” “FID”),        check = NULL)
ss$age.ipat4 <- cut(ss$age.ipat, breaks = c(0, 12, 18, 60, Inf),
            right = FALSE)
```

```
ss$age.ipat4 <- factor(ss$age.ipat4, levels = c("[18,60),"
                                        "[0,12),"
                                        "[12,18),"
                                        "[60,Inf)"))
ss$AgeGroup <- factor(ss$AgeGroup, levels = c("[18,Inf],"
                                        "[0,6),"
                                        "[6,12),"
                                        "[12,18)"))
ss$span.cat3 <- cut(ss$span.ind, breaks = c(0, 50, 90, Inf))
ss <- droplevels(ss)
```

Fit GLMM:

```
fit4 <- glmer(covid19 ~ antibody + age.ipat4 + hhs2 + sexi +
sexe+AgeGroup + span.cat3 + (1|FID), data = ss,
   control = glmerControl(optimizer = "bobyqa,"optCtrl = list(maxfun = 2e5)),
      family = binomial)
```

Odds ratios and Wald confidence intervals:

```
>m <- exp(cbind(odds = fixef(fit4), confint(fit4, parm = "β_," method = "Wald")))
```

Appendix Table 1. Severe acute respiratory syndrome coronavirus 2 IgG seropositivity rate at various time intervals after a positive reverse transcription PCR test for RNA from nasopharyngeal swab specimens in 634 participants from 473 households, southwest Germany, May–August, 2020

Time interval, d	No. participants, n = 634	No. seropositive participants, n = 575	Seropositivity rate, %
15–29	7	4	57.1
30–44	34	31	91.2
45–59	140	127	90.7
60–74	181	166	91.7
75–89	59	57	96.6
90–104	31	28	90.3
105–119	84	76	90.5
120–140	98	86	87.8

*All participants with a positive reverse transcription PCR test for severe acute respiratory syndrome coronavirus 2 RNA and with a positive or negative serologic result from 473 participating households before applying other inclusion or exclusion criteria were included in this analysis.

Appendix Table 2. Comparison of coronavirus disease–related symptoms in seropositive and seronegative index cases dependent on serostatus, southwest Germany, May–August, 2020

	No. (%)		
Symptoms	Index case-patients, n = 405*	Seropositive index case-patients, n = 363	Seronegative index case-patients, n = 42
Unknown	2 (0.49)	2 (0.55)	0
No	9 (2.22)	4 (1.10)	5 (11.9)
Yes	394 (97.2)	357 (98.3)	37 (88.1)
Fever†	226 (55.3)	212 (58.4)	14 (33.3)
Cough	237 (58.5)	213 (58.7)	24 (57.1)
Diarrhea	91 (22.5)	85 (23.4)	6 (14.3)
Dysgeusia	253 (62.5)	240 (66.1)	13 (31.0)
Hospitalization‡	22 (5.43)	20 (5.51)	2 (4.76)

*Percentages for all subgroups were calculated from 405 index cases.
†Presence or absence of fever, cough, diarrhea, and dysgeusia was known in all symptomatic index case-patients.
‡Hospitalization status was unknown in 1 symptomatic index case-patient (seropositive).

Appendix Table 3. Comparison of households with 2–3 and >4 household members, southwest Germany, May–August, 2020

No. household members	No. index cases, n = 405	No. seropositive exposed, n = 400	No. seronegative exposed, n = 820	Odds ratio (95% CI)*	p value
2–3	92	69	106	Referent	
>4	313	331	714	0.71 (0.51–1.01)	0.046

*Odds ratio and p value was calculated by Fisher exact test.

Appendix Table 4. Alternative regression model including 363 households with a severe acute respiratory syndrome coronavirus 2–seropositive index case-patient, southwest Germany, May–August, 2020

Predictor	Odds ratio (95% CI)*	p value
Age of index case-patient, >60 y	8.80 (1.11–69.9)	.039
Age of index case-patient, 12.0–17.9 y	1.43 (0.29–7.07)	.664
Age of index case-patient, 0.0–11.9 y	0.53 (0.04–1.83)	.621
Age of exposed household member, 12.0–17.9 y	0.41 (0.26–0.67)	<0.001
Age of exposed household member, 6.0–11.9 y	0.57 (0.36–0.92)	.022
Age of exposed household member, 0.0–5.9 y	0.35 (0.19–0.62)	<0.001
Female index case-patient	1.03 (0.59–1.81)	.911
Female exposed household member	1.11 (0.77–1.61)	.572
Household size, 4	0.50 (0.24–1.05)	.067
Household size, 5	0.78 (0.33–1.87)	.580
Household size, >6	0.45 (0.15–1.35)	.152

*Odds ratios and p values were calculated from a multivariable linear mixed-effects logistic regression model and the respective references were “adult index 18.0-59.9 years,” “adult exposed,” “male index,” “male exposed,” and “household size 2–3.”

Appendix Table 5. Alternative regression model including the predictor time interval (days) from the severe acute respiratory syndrome coronavirus 2 RNA-positive nasopharyngeal swab specimen collected in the index case-patient to the serologic assessment of 405 households, southwest Germany, May–August, 2020 *

Predictor	Odds ratio (95% CI)*	p value
Age of index case, >60 y	9.56 (1.24–73.67)	.030
Age of index case, 12.0–17.9 y	1.08 (0.26–4.46)	.911
Age of index case, 0.0–11.9 y	0.43 (0.05–3.78)	.448
Age of exposed household member, 12.0–17.9 y	0.39 (0.24–0.62)	<0.001
Age of exposed household member, 6.0–11.9 y	0.56 (0.35–0.89)	.015
Age of exposed household member, 0.0–5.9 y	0.33 (0.19–0.59)	<0.001
Female index case-patient	1.12 (0.65–1.93)	.680
Female exposed household member	1.09 (0.76–1.57)	.646
Household size, 4	0.44 (0.22–0.90)	.024
Household size, 5	0.62 (0.27–1.44)	.271
Household size, >6	0.36 (0.13–1.06)	.063
SARS-CoV-2–seropositive index case-patient	27.9 (8.41–92.33)	<0.001
Span from positive swab in index to study visit, 51–90 d	0.64 (0.26–1.62)	.348
Span from positive swab in index to study visit, 91–140 d	1.71 (0.64–4.56)	.282

*Odds ratios and p-values were calculated from a multivariable linear mixed-effects logistic regression model. Respective references were “adult index 18.0-59.9 years,” “adult exposed,” “male index,” “male exposed,” “household size 2–3,” “SARS-CoV-2 seronegative index case,” and “span from positive swab in index to study visit 30-50 days.”

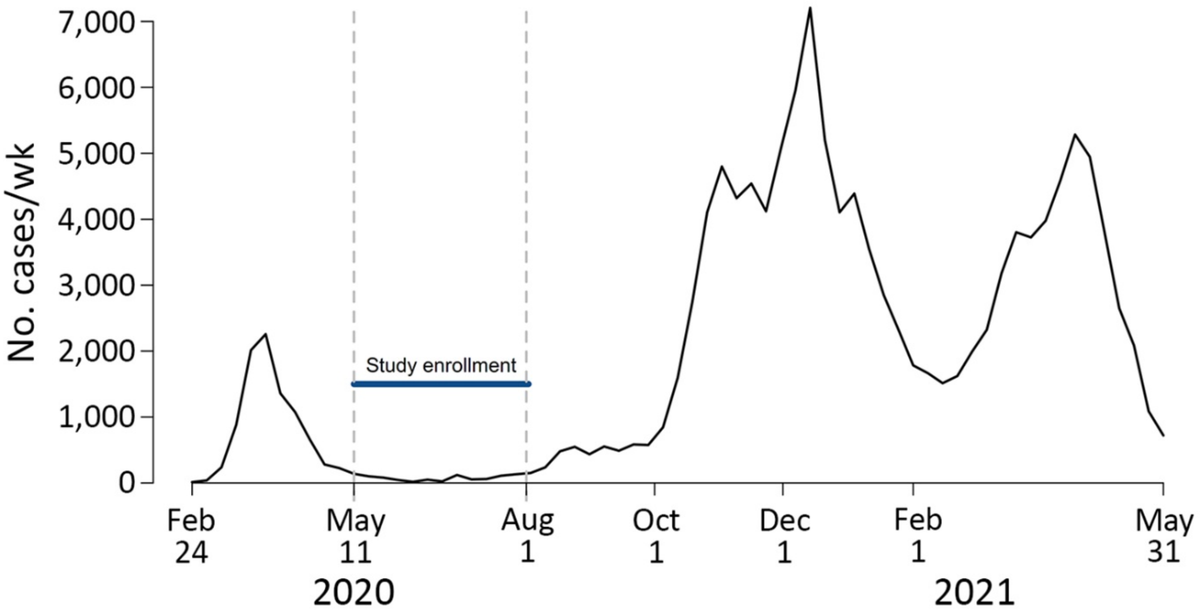

Appendix Figure. Weekly numbers of new SARS-CoV-2 infections registered by the local health authorities Alb-Donau/Ulm, Breisgau-Hochschwarzwald/Freiburg, Heidelberg/Rhein-Neckar, Karlsruhe, Mannheim, Neckar-Odenwald, Reutlingen, and Tübingen in the Federal State of Baden-Württemberg, Germany, by week of reporting during February 24, 2020–May 31, 2021 (n = 131.834). The study enrollment period was May 11, 2020–August 1, 2020. Data were retrieved and downloaded from the website of the Ministry of Social Affairs, Health and Integration, Federal State of Baden-Württemberg, Germany (*2*).

References

1. Gelman A, Hill J. Data analysis using regression and multilevel/hierarchical models. Cambridge: Cambridge University Press; 2007.

2. Ministry of Social Affairs, Health and Integration, Federal State of Baden-Württemberg, Germany. Coronavirus in Baden-Württemberg: Anzahl der Infizierten und der Todesfälle in Stadt-/Landkreisen [in German] [cited 2020 June 8]. https://sozialministerium.baden-wuerttemberg.de/fileadmin/redaktion/m-sm/intern/downloads/Downloads_Gesundheitsschutz/Tabelle_Coronavirus-Faelle-BW.xlsx
